# Supplementary material for: Expression Kinetics of Regulatory Genes Involved in the Vesicle Trafficking Processes Operating in Tomato Flower Abscission Zone Cells during Pedicel Abscission
Source: Life (Basel). 2020 Nov 6;10(11):273. doi: 10.3390/life10110273 (PMC7694662; doi:10.3390/life10110273)
Supplement: Supplementary file 1 [file life-10-00273-s001.zip › supplementary for XML/Figure S1&S2 .pdf]

life

MDPI

Supplementary materials of Expression Kinetics of Regulatory Genes Involved in the Vesicle Trafficking Processes Operating in Tomato Flower Abscission Zone Cells during Pedicel Abscission

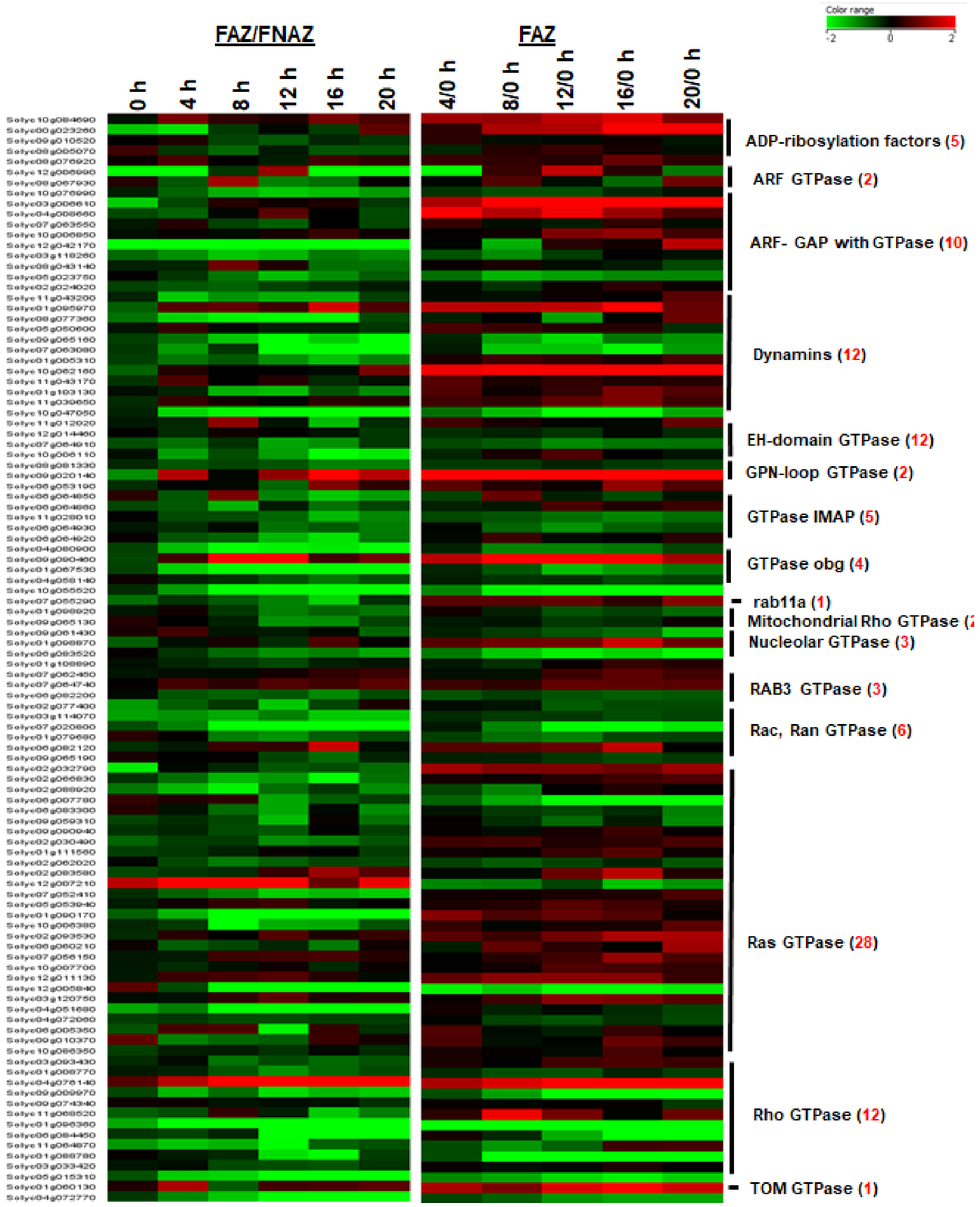

Figure S1. Heat map display of expression profiles of *GTPase*-related genes in the tomato flower AZ (FAZ) and in the proximal pedicel (NAZ) at various time points following abscission induction. The

expression profiles are presented as ratios of AZ-specificity (FAZ/NAZ), and as ratios of the expression in the FAZ at different time intervals relative to 0 h. The scale of the color range at the upper right of the figure represents the log2 ratio. The red numbers in parenthesis indicate the number of genes in each protein family. The genes in each category, their nomenclature, log2 ratios, SD, p values, and annotations of the tomato and the Arabidopsis genomes are presented in Supplementary Table S1.

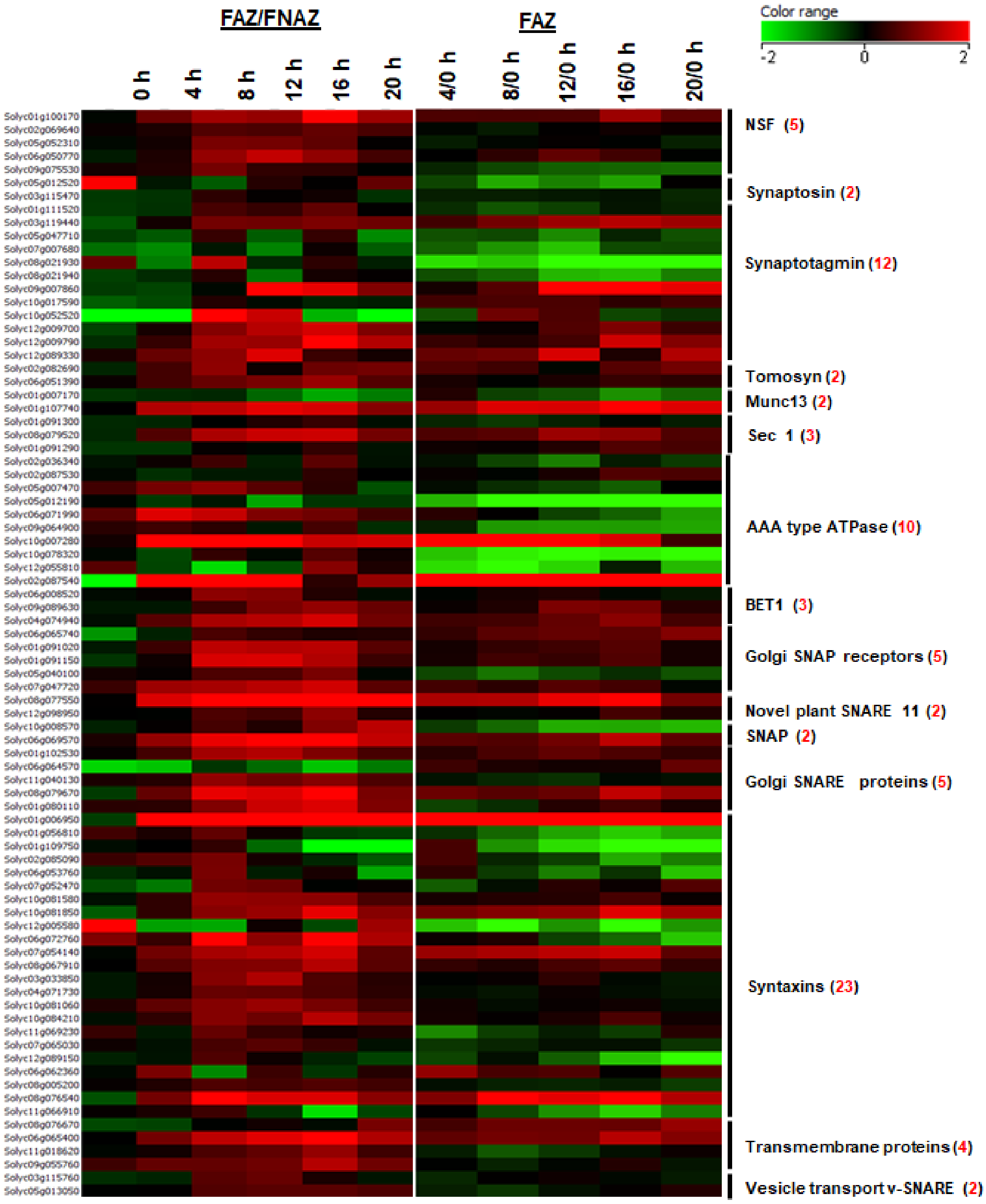

Figure S2. Heat map display of expression profiles of *SNARE* and *SNARE regulators*-related genes in the tomato flower AZ (FAZ) and in the proximal pedicel (NAZ) at various time points following abscission induction. All details are as presented in Supplementary Figure S1.

Publisher's Note: MDPI stays neutral with regard to jurisdictional claims in published maps and institutional affiliations.

© 2020 by the authors. Submitted for possible open access publication under the terms and conditions of the Creative Commons Attribution (CC BY) license (http://creativecommons.org/licenses/by/4.0/).
