# Supplementary material for: Expression Kinetics of Regulatory Genes Involved in the Vesicle Trafficking Processes Operating in Tomato Flower Abscission Zone Cells during Pedicel Abscission
Source: Life (Basel). 2020 Nov 6;10(11):273. doi: 10.3390/life10110273 (PMC7694662; doi:10.3390/life10110273)
Supplement: Supplementary file 1 [file life-10-00273-s001.zip › supplementary for XML/Figure S3.pdf]

life

Supplementary materials of Expression Kinetics of Regulatory Genes Involved in the Vesicle Trafficking Processes Operating in Tomato Flower Abscission Zone Cells during Pedicel Abscission

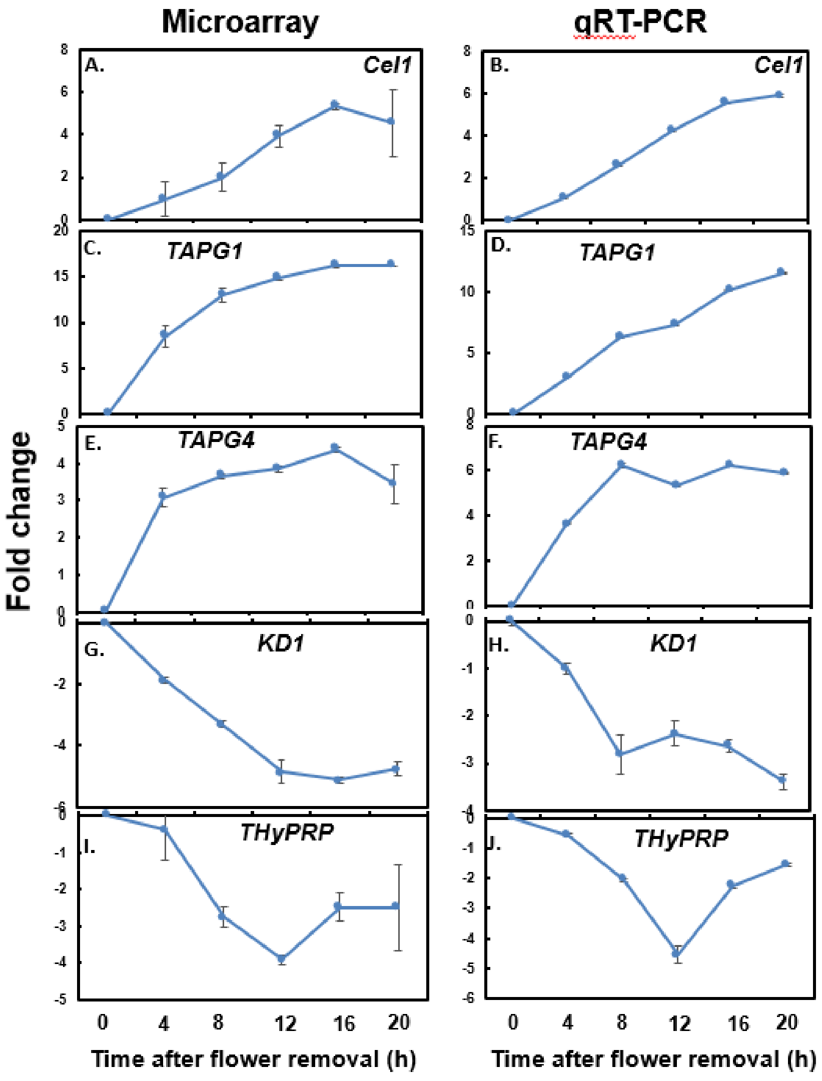

Figure S3. Validation of the microarray results (A, C, E, G, I) by qRT-PCR (B, D, F, H, J), of the kinetics of changes in expression patterns of selected genes in the FAZ of tomato (*Solanum lycopersicum*, cv. 'New Yorker') plants following abscission induction by flower removal. Expression levels were measured for tomato *Cellulase1* (*Cel1*) (ID - U13054/Solyc08g081620) (A, B); *Tomato Abscission Polygalacturonase1 (TAPG1) (ID - U23053/Solyc02g067630);* (C, D); *TAPG4* (ID - U70481/ Solyc12g096750) (E, F); *knotted1-like homeobox protein (KD1)* (ID - AF375969/ Solyc06g072480) (G, H); and *THyPRP* (ID - X57076/Solyc07g043000) (I, J). The data indicate the fold change relative to time

zero. The relative quantification of the gene expression level in the qRT-PCR assay was determined by the comparative C_T method $2^{-\Delta\Delta C}T$ using *ACTIN* as a reference gene, and then calculating the fold change value. The ΔCT values for each gene were compared to the zero time ΔCT value for that gene to generate the $2^{-\Delta\Delta C}T$ values. The microarray and qRT-PCR analyses were performed with different samples taken from independent biological replicates of two separate experiments. The results are means of two biological replicates ± SD. The list of primers and their sequences used in the qRT-PCR assay is presented in Table S3.

Publisher's Note: MDPI stays neutral with regard to jurisdictional claims in published maps and institutional affiliations.

© 2020 by the authors. Submitted for possible open access publication under the terms and conditions of the Creative Commons Attribution (CC BY) license (http://creativecommons.org/licenses/by/4.0/).
