# Supplementary material for: Expression Kinetics of Regulatory Genes Involved in the Vesicle Trafficking Processes Operating in Tomato Flower Abscission Zone Cells during Pedicel Abscission
Source: Life (Basel). 2020 Nov 6;10(11):273. doi: 10.3390/life10110273 (PMC7694662; doi:10.3390/life10110273)

Supplementary Table S1. Summary of gene nomenclature, expression timing and annotation details for GTPase-related tomato genes, which are presented in the Supplementary Figure S1.

				AZ						NAZ						AZ/NAZ					
S No.	Category	Locus Id	Description	0h_AZ	4h_AZ	8h_AZ	12h_AZ	16h_AZ	20h_AZ	0h_NAZ	4h_NAZ	8h_NAZ	12h_NAZ	16h_NAZ	20h_NAZ	0h_AZ/NAZ	4h_AZ/NAZ	8h_AZ/NAZ	12h_AZ/NAZ	16h_AZ/NAZ	20h_AZ/NAZ
1	GTPase	Solyc10g084690	ADP-ribosylation factor GTPase	0.000	1.236	1.168	1.473	1.763	0.977	0.000	0.123	0.603	0.904	0.208	-0.038	0.078	1.408	1.502	1.361	2.248	1.304
2	GTPase	Solyc00g023260	ADP-ribosylation factor GTPase	0.000	0.350	1.333	1.379	1.995	1.913	0.000	0.605	0.449	-0.019	0.598	-0.291	-1.341	-1.334	0.689	1.094	0.867	1.473
3	GTPase	Solyc09g010520	ADP-ribosylation factor GTPase	0.000	0.321	-0.105	-0.064	0.112	-0.158	0.000	0.109	0.426	0.470	-0.049	0.066	0.257	0.699	0.616	0.454	1.059	0.320
4	GTPase	Solyc08g005070	ADP-ribosylation factor GTPase-	0.000	-0.281	0.276	0.432	0.161	-0.105	0.000	0.466	1.386	0.897	0.695	0.638	0.712	0.067	0.338	0.966	0.675	0.093
5	GTPase	Solyc08g076920	ADP-ribosylation factor GTPase-activatin	0.000	0.423	0.372	0.285	0.787	0.348	0.000	-0.082	0.425	0.411	-0.093	-0.137	0.343	1.051	1.157	0.926	1.840	1.083
6	GTPase	Solyc12g006990	Arf GTPase activating protein	0.000	-2.506	0.430	1.529	0.305	-0.943	0.000	-0.639	-1.485	-2.174	-0.508	-0.031	-2.044	-3.719	0.724	2.320	-0.685	-2.702
7	GTPase	Solyc08g067930	Arf GTPase activating protein	0.000	-0.058	0.639	-0.095	-0.819	0.867	0.000	0.759	-0.057	0.762	-0.103	0.879	0.521	-0.196	2.411	0.143	0.473	0.785
8	GTPase	Solyc10g076990	Arf-GAP domain and FG repeats-containi	0.000	-0.399	-0.623	-0.619	-0.313	-0.125	0.000	0.114	0.507	0.535	0.470	0.647	0.000	-0.294	-0.233	-0.437	-0.147	-0.481
9	GTPase	Solyc03g006610	Arf-GAP with GTPase, ANK repeat	0.000	1.338	2.733	2.880	3.528	2.568	0.000	0.308	0.814	0.992	1.257	1.153	-1.368	-0.072	1.416	1.235	1.556	0.282
11	GTPase	Solyc04g008660	Arf-GAP with GTPase, ANK repeat	0.000	2.121	1.412	1.903	1.200	0.590	0.000	1.949	0.819	0.477	0.165	0.404	-0.330	-0.293	1.099	1.844	1.337	0.157
12	GTPase	Solyc07g063550	Arf-GAP with GTPase, ANK repeat and PH	0.000	0.403	-0.101	-0.223	0.388	-0.097	0.000	-0.070	0.235	0.165	-0.232	0.055	0.061	0.772	0.637	0.410	1.334	0.188
13	GTPase	Solyc10g006850	Arf-GAP with GTPase, ANK repeat	0.000	0.030	0.110	0.969	1.131	0.502	0.000	-0.179	0.076	0.484	0.119	0.046	0.120	0.545	1.038	1.318	1.762	0.841
14	GTPase	Solyc12g042170	Arf-GAP with GTPase, ANK repeat	0.000	-0.029	-1.375	0.299	0.140	1.453	0.000	0.052	0.015	1.388	0.866	2.484	-2.102	-1.939	-2.639	-2.484	-2.199	-2.892
15	GTPase	Solyc03g118260	Arf-GAP with GTPase, ANK repeat	0.000	-0.643	-1.105	-0.465	0.017	-0.112	0.000	-0.331	-0.401	-0.058	-0.308	-0.065	-0.598	-0.660	-0.422	-0.287	0.372	-0.359
16	GTPase	Solyc08g043140	Arf-GAP with GTPase, ANK repeat	0.000	0.060	0.197	-0.095	-0.195	-0.560	0.000	0.052	-0.937	-0.773	-0.045	-0.151	-0.012	0.197	1.980	1.369	0.463	-0.153
17	GTPase	Solyc05g023750	Arf-GAP with GTPase, ANK repeat	0.000	-0.870	-1.410	-1.244	-1.059	-1.060	0.000	-0.463	-0.500	0.064	-0.406	-0.513	0.252	0.063	0.225	-0.329	0.234	0.009
18	GTPase	Solyc02g024020	Arf-GAP with GTPase, ANK repeat	0.000	-0.070	-0.388	0.089	0.341	0.084	0.000	0.081	-0.174	-0.110	-0.262	-0.447	-0.279	-0.207	0.378	0.637	0.954	0.527
19	GTPase	Solyc11g043200	Dynamin, GTPase region	0.000	-0.067	-0.107	0.102	0.038	0.701	0.000	1.097	0.729	0.990	0.665	0.715	-0.034	-1.112	-0.006	-0.212	-0.033	0.234
20	GTPase	Solyc01g095970	Dynamin 2 , GTPase region	0.000	1.454	1.438	1.498	2.263	0.803	0.000	0.012	-0.160	0.057	-0.662	-0.838	-0.507	1.127	1.891	1.608	3.011	1.310
21	GTPase	Solyc08g077360	Dynamin 2 , GTPase region	0.000	-0.225	-0.017	-1.258	-0.034	0.817	0.000	0.559	0.993	0.811	0.919	0.448	-0.653	-1.188	-0.778	-1.793	-0.963	0.170
22	GTPase	Solyc05g050600	Dynamin 2 , GTPase region	0.000	0.564	0.416	0.257	0.282	-0.348	0.000	-0.025	0.322	0.167	-0.098	-0.311	0.082	0.877	1.057	0.882	1.078	0.309
23	GTPase	Solyc09g065160	Dynamin family protein, GTPase region	0.000	-0.267	-1.264	-1.710	-0.913	-1.155	0.000	0.111	-0.201	0.058	-0.251	0.171	-0.255	-0.405	-0.407	-1.243	-0.265	-1.284
24	GTPase	Solyc07g063080	Dynamin family protein GTPase region	0.000	-0.193	-1.542	-1.520	-1.919	-1.219	0.000	0.533	-1.342	0.043	0.401	1.072	-0.199	-0.737	0.729	-1.094	-2.075	-2.148
25	GTPase	Solyc01g005310	Dynamin like protein GTPase region	0.000	0.186	0.494	0.376	0.199	0.541	0.000	0.602	0.705	0.846	0.751	0.847	-0.249	-0.451	0.436	-0.004	-0.175	-0.289
26	GTPase	Solyc10g062160	Dynamin like protein , GTPase region	0.000	1.884	2.243	1.967	3.004	2.827	0.000	1.015	1.635	1.197	2.012	0.906	-0.530	0.738	1.138	1.152	1.266	1.654
27	GTPase	Solyc11g043170	Dynamin-2 , GTPase region	0.000	0.655	0.212	0.361	0.234	0.296	0.000	-0.395	-0.240	-0.491	-0.323	-0.216	-0.170	1.079	1.130	1.436	1.014	0.615
28	GTPase	Solyc01g103130	Dynamin-2A , GTPase region	0.000	0.617	0.094	0.395	0.961	0.626	0.000	0.812	1.337	1.583	1.167	1.282	0.130	0.202	-0.271	-0.332	0.691	-0.330
29	GTPase	Solyc11g039650	Dynamin-2A , GTPase region	0.000	0.485	0.372	0.723	0.973	0.469	0.000	-0.288	-0.142	0.216	-0.182	-0.404	-0.098	0.887	1.284	1.118	1.675	1.031
30	GTPase	Solyc10g047050	Dynamin-like protein , GTPase region	0.000	-0.862	-1.455	-2.121	-2.017	-1.300	0.000	0.528	0.200	0.233	0.046	0.450	-0.038	-1.202	-0.819	-1.606	-1.468	-1.467
31	GTPase	Solyc11g012020	EH-domain-containing protein 1 , GTPas	0.000	0.506	0.187	-0.030	-0.046	0.784	0.000	0.478	-1.095	-0.262	0.723	1.119	0.047	0.166	2.246	0.991	-0.138	0.401
32	GTPase	Solyc12g014460	EH-domain-containing protein 1 , GTPas	0.000	-0.359	-0.250	-0.392	-0.046	-0.323	0.000	-0.029	-0.351	-0.577	-0.346	-0.098	0.252	0.152	1.254	1.167	1.193	0.302
33	GTPase	Solyc07g064910	EH-domain-containing protein 1 , GTPas	0.000	-0.565	-0.715	-1.081	-0.926	-0.891	0.000	-0.291	-0.943	-0.585	-0.875	-1.210	-0.370	-0.403	0.763	-0.139	0.215	0.316
34	GTPase	Solyc10g006110	EH-domain-containing protein 1, GTPase	0.000	-0.281	0.182	0.596	0.010	-0.148	0.000	0.760	0.654	1.555	2.348	1.409	0.110	-0.776	0.533	-0.079	-1.567	-1.109
35	GTPase	Solyc08g081330	GPN-loop GTPase 1	0.000	-0.449	-0.442	-0.269	-0.430	-0.516	0.000	-0.134	-0.235	-0.126	-0.208	-0.063	-0.085	-0.192	0.603	0.485	0.323	-0.274
36	GTPase	Solyc09g020140	GPN-loop GTPase 3	0.000	2.124	2.099	2.273	3.481	2.376	0.000	-0.058	1.149	0.195	-0.554	-0.028	-0.859	1.869	1.098	2.256	4.211	2.150
38	GTPase	Solyc06g053190	GTPase activating-like protein	0.000	0.688	0.296	0.093	1.134	0.613	0.000	0.356	0.028	0.407	-0.689	-0.332	-0.123	0.451	1.074	0.359	2.342	1.083
39	GTPase	Solyc06g064850	GTPase IMAP family member 7	0.000	-0.503	0.793	-0.239	-0.555	-0.132	0.000	0.468	0.745	0.930	0.852	1.144	0.546	-0.200	2.066	0.126	-0.234	-0.496
40	GTPase	Solyc06g064860	GTPase IMAP family member 7	0.000	-0.252	-0.172	0.555	0.252	0.454	0.000	-0.161	0.499	-0.032	0.153	0.124	-0.317	-0.227	-0.270	0.937	0.449	0.207
41	GTPase	Solyc11g028010	GTPase IMAP family member 7	0.000	-0.566	-0.653	-0.850	-1.032	-0.857	0.000	-0.010	-0.087	-0.221	-0.043	-0.026	0.243	-0.100	0.543	0.341	-0.121	-0.322
42	GTPase	Solyc06g064930	GTPase IMAP family member 7	0.000	-0.035	-0.784	-1.153	-0.753	-0.558	0.000	0.134	-0.947	-0.628	-0.685	-0.003	0.061	0.126	1.072	0.273	0.635	-0.242
43	GTPase	Solyc06g064920	GTPase IMAP family member 7	0.000	-0.048	0.457	-0.076	-0.015	0.302	0.000	0.604	1.194	1.165	1.258	1.022	0.288	-0.195	0.349	-0.287	-0.447	-0.219
45	GTPase	Solyc04g080900	GTPase obg	0.000	-0.270	-1.020	-1.020	-0.916	-0.506	0.000	0.683	0.741	0.184	-0.067	0.839	-0.267	-1.000	-1.158	-0.756	-0.490	-1.359
46	GTPase	Solyc09g090460	GTPase obg	0.000	1.440	2.883	2.841	2.054	1.258	0.000	0.394	-0.019	-0.597	0.497	-0.371	-0.319	0.971	3.449	3.842	1.830	1.603
47	GTPase	Solyc01g067530	GTPase obg	0.000	-0.278	-0.666	-1.475	-1.203	-0.945	0.000	0.808	1.126	0.875	0.430	1.066	-0.277	-1.154	-1.206	-1.836	-1.290	-2.012
48	GTPase	Solyc04g058140	GTPase obg	0.000	-0.230	-0.393	-0.351	-0.591	-0.506	0.000	0.106	0.111	0.208	-0.044	-0.012	0.332	0.222	0.767	0.500	0.419	0.118
50	GTPase	Solyc10g055520	GTP-binding RHO-like protein Ras small	0.000	-1.045	-1.874	-2.953	-2.856	-1.973	0.000	-0.105	0.073	-0.103	-0.246	-0.237	-0.096	-0.749	-1.118	-2.061	-2.012	-1.486
51	GTPase	Solyc07g055290	LES245570 Rab11 GTPase	0.000	0.692	0.704	0.795	0.353	1.046	0.000	0.207	0.513	0.764	0.652	0.414	-0.637	0.060	0.421	0.126	-0.324	0.364
52	GTPase	Solyc01g098920	Mitochondrial Rho GTPase 1	0.000	0.128	-0.245	-0.637	-0.496	-0.805	0.000	-0.205	-0.024	-0.005	-0.360	-0.285	0.066	0.607	0.705	0.157	0.555	-0.181
53	GTPase	Solyc09g065130	Mitochondrial Rho GTPase 1	0.000	-0.186	-0.150	-0.433	-0.335	0.093	0.000	0.096	0.359	0.801	0.600	0.644	0.481	0.473	0.904	0.008	0.219	0.209
54	GTPase	Solyc09g061430	nuclear GTPase DQ304482	0.000	-0.247	-0.454	-0.666	-0.829	-1.561	0.000	-0.602	-0.093	-0.422	-1.032	-0.757	0.424	0.995	0.977	0.946	1.262	-0.040
55	GTPase	Solyc01g098870	Nucleolar GTPase	0.000	0.661	0.851	0.870	1.595	0.821	0.000	0.021	-0.042	0.292	-0.185	-0.180	-0.483	0.374	1.319	0.816	1.931	0.761
56	GTPase	Solyc06g083520	Nucleolar GTP-binding protein 2	0.000	-0.950	-1.567	-1.864	-1.662	-1.949	0.000	-0.573	-0.760	-0.930	-1.129	-0.838	0.259	0.084	0.309	0.022	0.343	-0.563
57	GTPase	Solyc01g108890	Potential GTPase activation protein	0.000	-0.183	-0.372	0.053	0.416	0.277	0.000	-0.031	-0.096	0.316	0.086	0.117	0.123	0.195	0.728	0.583	1.094	0.561
58	GTPase	Solyc07g062450	RAB3 GTPase activating protein subunit	0.000	-0.101	-0.121	0.453	0.706	0.509	0.000	-0.198	-0.209	-0.056	-0.295	-0.333	0.063	0.375	1.051	1.290	1.695	1.180
59	GTPase	Solyc07g064740	Rab3 GTPase-activating protein catalytic	0.000	0.471	0.351	0.757	0.749	0.689	0.000	-0.047	0.001	-0.049	-0.207	-0.231	0.243	0.982	1.463	1.767	1.826	1.431
60	GTPase	Solyc06g082200	Rab3 GTPase-activating protein catalytic	0.000	-0.652	-0.461	-0.642	-0.739	-0.683	0.000	-0.085	0.248	-0.198	-0.222	-0.402	0.162	-0.189	0.367	0.460	0.265	0.146
61	GTPase	Solyc02g077400	Rac-like GTP binding protein	0.000	-0.079	-0.143	-0.725	-0.579	-0.570	0.000	-0.609	-0.984	-0.927	-1.915	-2.274	-0.975	-0.264	0.698	-0.414	0.783	0.940
62	GTPase	Solyc03g114070	Rac-like GTP-binding protein 4	0.000	-0.320	-0.456	-0.433	-0.608	-0.521	0.000	-0.490	-0.373	-0.904	-0.781	-0.467	-1.035	-0.666	-0.200	0.138	-0.239	-0.834
63	GTPase	Solyc07g020800	Rac-like GTP-binding protein 4 e	0.000	-0.182	-1.036	-1.960	-2.129	-1.813	0.000	0.131	0.436	1.042	0.688	1.148	-0.377	-0.536	-0.993	-2.559	-2.598	-3.059
64	GTPase	Solyc01g079680	Ran GTPase activating protein	0.000	-0.494	-0.858	-1.002	-0.546	-0.502	0.000	-0.252	0.394	0.238	-0.005	0.172	0.523	0.511	0.157	-0.004	0.607	0.174
65	GTPase	Solyc06g082120	Ran GTPase binding protein	0.000	0.644	0.666	0.789	1.490	0.027	0.000	0.401	-0.170	-0.149	-0.919	-0.564	-0.120	0.292	1.561	1.511	2.909	0.641
66	GTPase	Solyc09g065190	Ran GTPase-activating protein 1	0.000	-0.244	-0.255	-0.418	-0.559	-0.420	0.000	-0.103	-0.131	0.087	-0.033	-0.195	0.405	0.470	1.170	0.624	0.500	0.467
67	GTPase	Solyc02g032790	Ras GTPase-activating protein-binding p	0.000	1.259	0.999	0.972	0.829	1.192	0.000	-0.616	-0.589	-0.418	-0.766	-0.069	-1.630	0.420	0.838	0.462	0.725	-0.092
68	GTPase	Solyc02g066830	Ras GTPase-activating protein-binding p	0.000	0.168	0.092	0.300	0.489	0.593	0.000	0.600	1.019	0.893	2.116	0.849	-0.158	-0.377	-0.266	-0.038	-1.665	-0.151
69	GTPase	Solyc02g088920	Ras GTPase-activating protein-binding p	0.000	-0.533	-1.016	-0.018	0.255	-0.016	0.000	-0.003	-0.010	-0.228	-0.660	-0.037	-0.690	-0.968	-0.777	0.290	0.905	-0.368
70	GTPase	Solyc06g007780	Ras GTPase-activating protein-binding p	0.000	-0.779	-1.323	-2.511	-2.487	-2.879	0.000	-0.679	-1.259	-0.997	-1.834	-2.125	0.622	0.709	1.482	-0.149	0.618	0.254
71	GTPase	Solyc06g083300	Ras GTPase-activating protein-binding p	0.000	-0.138	-0.441	-0.560	-0.330	-1.007	0.000	0.294	0.677	0.882	-0.500	0.097	0.515	0.339	0.314	-0.150	1.271	-0.359
72	GTPase	Solyc09g059310	Ras GTPase-activating protein-binding p	0.000	0.061	-0.346	-0.821	-0.202	-1.073	0.000	-0.131	-0.316	-0.049	-1.298	-0.972	-0.297	0.132	0.586	-0.331	1.402	-0.146
73	GTPase	Solyc09g090940	Ras GTPase-activating protein-binding p	0.000	0.066	-0.095	0.182	0.449	0.065	0.000	-0.075	0.085	0.263	-0.406	-0.084	-0.148	0.189	0.547	0.470	1.309	0.247
75	GTPase	Solyc02g030490	Ras-GTPase-activating protein SH3-doma	0.000	0.454	0.516	0.232	0.350	0.558	0.000	0.212	0.181	0.418	0.147	0.230	-0.544	0.007	0.684	0.040	0.363	0.084
76	GTPase	Solyc01g111560	Ras-like GTP-binding protein RHO	0.000	0.141	0.080	0.408	0.650	0.100	0.000	0.045	-0.155	-0.348	-0.297	-0.141	-0.258	0.066	0.882	1.226	1.329	0.268
77	GTPase	Solyc02g062020	Ras-like GTP-binding protein RHO	0.000	-0.500	-0.710	-0.528	-0.266	-0.674	0.000	-0.037	0.148	-0.059	-0.288	-0.396	0.191	-0.089	0.179	0.397	0.813	0.149
78	GTPase	Solyc02g083580	Ras-like GTP-binding protein RHO (AHR	0.000	-0.062	-0.073	0.819	1.470	0.266	0.000	-0.228	-0.338	-0.350	-0.752	-1.232	-0.320	0.053	0.796	1.557	2.530	1.415
79	GTPase	Solyc12g007210	Ras-like GTP-binding protein RHO	0.000	-1.047	-0.976	-0.508	-1.535	-1.162	0.000	-1.932	-1.926	-1.347	-1.306	-1.745	1.687	2.744	3.552	3.297	2.120	2.563
80	GTPase	Solyc07g052410	Ras-related protein Rab-18	0.000	0.117	0.136	0.200	0.234	0.564	0.000	0.512	0.815	1.227	0.858	1.423	-0.112	-0.296	0.090	-0.456	-0.114	-0.870
81	GTPase	Solyc05g053940	Ras-related protein Rab-1A	0.000	0.199	0.284	0.679	0.493	0.175	0.000	-0.189	-0.362	-0.201	-0.042	-0.308	0.012	0.614	1.523	1.610	1.173	0.759
82	GTPase	Solyc01g090170	Ras-related protein Rab-1A	0.000	1.037	0.562	0.788	0.633	0.086	0.000	1.611	1.863	2.178	2.259	1.743	-0.517	-0.928	-1.024	-1.131	-1.544	-1.683
83	GTPase	Solyc10g006380	Ras-related protein Rab-21	0.000	0.410	-0.042	0.198	0.179	0.687	0.000	0.609	2.689	1.069	0.736	0.803	-0.060	0.017	-2.942	-0.219	0.008	0.093
84	GTPase	Solyc02g093530	Ras-related protein Rab-2-A	0.000	0.696	0.436	0.876	1.337	1.312	0.000	0.053	0.068	-0.248	0.281	0.372	-0.095	0.765	1.140	1.781	1.571	1.142
85	GTPase	Solyc06g060210	Ras-related protein Rab-2-A	0.000	-0.217	0.737	0.341	-0.012	1.228	0.000	0.454	1.276	0.567	0.463	0.852	0.323	-0.250	0.594	0.870	0.387	0.856
86	GTPase	Solyc07g056150	Ras-related protein Rab-2-A	0.000	0.018	0.285	0.508	1.172	0.581	0.000	-0.006	-0.397	-0.366	-0.036	-0.123	0.054	0.288	1.597	1.638	1.885	1.014
87	GTPase	Solyc10g007700	Ras-related protein Rab-2-A	0.000	-0.017	0.138	0.597	0.537	0.379	0.000	0.125	0.049	0.126	0.204	-0.121	0.055	0.132	1.020	1.255	1.019	0.817
88	GTPase	Solyc12g011130	Ras-related protein Rab-2-A	0.000	0.746	1.058	1.049	1.037	0.423	0.000	0.107	0.312	0.060	0.095	0.102	0.044	0.912	1.663	1.774	1.618	0.641
89	GTPase	Solyc12g005840	Ras-related protein Rab-5C	0.000	-1.911	-1.581	-2.039	-2.390	-1.852	0.000	-0.654	1.184	1.281	1.469	0.610	0.921	-0.085	-0.943	-1.568	-2.279	-1.287
91	GTPase	Solyc03g120750	Ras-related protein Rab-7	0.000	0.090	0.478	1.064	0.963	0.691	0.000	0.101	-0.003	0.061	0.167	0.070	0.140	0.345	1.507	1.859	1.566	1.027
92	GTPase	Solyc04g051680	Ras-related protein Rab-7	0.000	0.139	-0.258	-0.062	-0.316	-0.518	0.000	-0.223	0.701	0.451	0.847	0.716	-1.088	-0.510	-1.182	-0.897	-1.604	-2.058
93	GTPase	Solyc04g072060	Ras-related protein Rab-7a	0.000	-0.033	-0.521	-0.644	-0.362	-0.559	0.000	-0.117	-0.421	-0.720	-0.805	-0.772	-0.242	0.059	0.550	0.549	0.835	0.248
94	GTPase	Solyc06g005350	Ras-related protein Rab-8A	0.000	0.618	-0.083	-0.226	0.409	0.011	0.000	-0.513	-1.418	0.643	-1.137	-0.459	-0.406	1.004	1.757	-0.789	1.739	0.358
95	GTPase	Solyc09g010370	Ras-related protein Rab-8A	0.000	0.589	-0.121	-0.031	0.798	0.444	0.000	1.759	1.183	1.056	0.694	0.740	0.990	-0.528	0.331	0.412	1.771	0.886
96	GTPase	Solyc10g086350	Ras-related protein Rab-8A	0.000	0.176	0.055	-0.075	0.556	0.029	0.000	-0.127	-0.262	-0.372	-0.370	-0.382	-0.256	0.239	0.926	0.759	1.283	0.401
97	GTPase	Solyc03g093430	Rho GTPase activating protein 1	0.000	0.210	0.000	0.486	0.588	0.442	0.000	-0.082	0.557	0.523	0.599	0.538	-0.100	0.397	0.243	0.582	0.520	0.076
98	GTPase	Solyc01g008770	Rho GTPase activating protein 12	0.000	-0.325	-0.622	-0.647	-0.268	-0.479	0.000	-0.283	-0.165	0.107	-0.129	-0.306	0.030	0.213	0.473	-0.001	0.527	0.152
99	GTPase	Solyc04g076140	Rho GTPase activating protein 12	0.000	1.325	1.715	4.205	5.012	4.463	0.000	0.534	0.146	0.140	0.658	0.082	0.881	1.905	3.321	5.714	5.815	5.504
100	GTPase	Solyc09g009970	Rho GTPase activating protein 2	0.000	-0.593	-1.244	-2.433	-2.527	-2.237	0.000	0.152	-1.051	-1.486	-2.058	-1.505	-0.311	-0.972	0.286	-0.595	-0.119	-0.851
101	GTPase	Solyc09g074340	Rho GTPase activating protein 2	0.000	0.041	-0.044	0.028	0.048	-0.444	0.000	0.010	0.186	0.057	-0.168	-0.189	0.345	0.589	0.984	1.038	1.190	0.354
102	GTPase	Solyc11g068520	Rho GTPase activating protein 2	0.000	0.330	1.873	0.907	-0.026	0.827	0.000	0.222	0.733	0.376	0.380	0.822	-0.508	-0.125	1.519	0.898	-0.263	-0.240
103	GTPase	Solyc01g096360	Rho GTPase activating protein 2	0.000	-2.953	-3.615	-4.728	-4.534	-4.377	0.000	-0.754	-0.699	-0.924	-1.245	-0.903	-1.091	-3.055	-3.121	-4.116	-3.729	-4.248
104	GTPase	Solyc06g084450	Rho GTPase activating protein 2	0.000	0.162	-0.317	-1.363	-2.594	-2.273	0.000	0.188	-0.507	0.302	0.113	0.843	-0.167	-0.057	0.856	-1.172	-2.253	-2.483
105	GTPase	Solyc11g064870	Rho GTPase activating protein 22	0.000	-0.570	-1.175	-0.777	0.468	0.522	0.000	-0.221	-0.958	-0.137	-0.379	-0.248	-0.951	-1.064	-0.257	-0.834	0.554	0.102
106	GTPase	Solyc01g088780	Rho-related GTP-binding protein RhoC	0.000	-0.587	-2.068	-2.809	-3.410	-2.791	0.000	-0.498	-1.766	-1.020	-1.903	-2.656	0.251	0.271	0.814	-0.625	-0.652	0.253
107	GTPase	Solyc03g033420	Rho-related GTP-binding protein RhoC	0.000	0.070	-0.117	-0.072	0.228	-0.014	0.000	-0.011	0.296	0.179	0.253	-0.003	0.068	0.382	0.564	0.539	0.676	0.329
108	GTPase	Solyc05g015310	Ribosome-associated GTPase	0.000	-1.049	-1.302	-1.691	-1.891	-1.612	0.000	-0.072	-0.300	-0.274	-0.406	-0.311	-0.401	-1.131	-0.529	-1.104	-1.265	-1.357
109	GTPase	Solyc01g060130	TOMGTPASE Mill. GTPase	0.000	1.328	0.941	1.731	2.077	1.603	0.000	0.178	1.841	1.278	1.286	0.923	0.463	1.829	0.429	1.635	1.888	1.433
110	GTPase	Solyc04g072770	tRNA modification GTPase mnmE	0.000	-0.536	-0.736	-1.166	-1.296	-1.293	0.000	0.091	0.370	0.254	-0.097	0.208	-0.190	-0.601	-0.428	-0.893	-0.763	-1.333

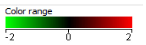

Supplement: Supplementary file 1 [file life-10-00273-s001.zip › supplementary for XML/Supplementary Table S1.pdf]
