# Supplementary material for: Expression Kinetics of Regulatory Genes Involved in the Vesicle Trafficking Processes Operating in Tomato Flower Abscission Zone Cells during Pedicel Abscission
Source: Life (Basel). 2020 Nov 6;10(11):273. doi: 10.3390/life10110273 (PMC7694662; doi:10.3390/life10110273)

Supplementary Table S2. Summary of gene nomenclature, expression timing and annotation details for SNARE-related tomato genes, which are presented in the Supplementary Figure S2.

				AZ						NAZ						AZ/NAZ					
S.No	Category	Locus Id	Description	0h_AZ	4h_AZ	8h_AZ	12h_AZ	16h_AZ	20h_AZ	0h_NAZ	4h_NAZ	8h_NAZ	12h_NAZ	16h_NAZ	20h_NAZ	0h_AZ/NAZ	4h_AZ/NAZ	8h_AZ/NAZ	12h_AZ/NAZ	16h_AZ/NAZ	20h_AZ/NAZ
1	NSF	Solyc01g100170	Soluble NSF Attachment Protein (SNAP) Receptor (SNARE)	0.000	0.612	0.595	0.611	1.239	0.729	0.000	-0.064	0.145	0.108	-0.166	-0.305	-0.046	0.840	1.270	1.170	1.980	1.240
2	NSF	Solyc02g069640	Gamma-soluble NSF attachment protein	0.000	-0.039	-0.204	0.022	0.140	0.058	0.000	0.040	0.140	0.233	0.073	-0.157	0.084	0.229	0.637	0.596	0.783	0.568
3	NSF	Solyc05g052310	Alpha-soluble NSF attachment protein	0.000	-0.212	-0.046	0.061	-0.013	-0.239	0.000	-0.194	-0.167	-0.184	-0.193	-0.071	-0.052	0.144	0.947	0.915	0.745	0.046
4	NSF	Solyc06g050770	Alpha-soluble NSF attachment protein	0.000	0.000	0.358	0.764	0.512	-0.008	0.000	-0.208	-0.132	-0.248	-0.183	-0.474	-0.189	0.232	1.174	1.537	1.140	0.535
5	NSF	Solyc09g075530	Soluble NSF Attachment Protein (SNAP) Receptor (SNARE)	0.000	-0.271	-0.548	-0.729	-0.781	-0.819	0.000	-0.132	-0.405	-0.203	-0.342	-0.367	0.169	0.255	0.918	0.388	0.369	-0.007
6	Synaptosin	Solyc05g012520	Synaptosomal-associated protein 30	0.000	-0.525	-1.335	-1.002	-1.264	0.028	0.000	1.095	1.570	0.773	0.709	1.333	1.993	-0.168	-0.696	0.289	0.017	0.786
7	Synaptosin	Solyc03g115470	Synaptosomal-associated protein 33	0.000	-0.223	-0.230	-0.185	-0.128	-0.290	0.000	-0.053	-0.089	0.053	-0.047	-0.077	-0.431	-0.378	0.349	0.035	0.127	-0.367
8	Synaptotagmin	Solyc01g111520	Synaptotagmin	0.000	-0.344	-0.651	-0.498	-0.152	-0.266	0.000	-0.199	-0.771	-0.652	-0.728	-0.471	-0.443	-0.374	0.557	0.421	0.761	0.028
9	Synaptotagmin	Solyc03g119440	Plant synaptotagmin	0.000	0.511	0.910	1.237	1.429	1.231	0.000	-0.079	0.218	0.431	0.430	-0.017	-0.650	0.153	0.913	0.880	0.977	0.859
10	Synaptotagmin	Solyc05g047710	Plant synaptotagmin	0.000	-0.623	-0.566	-1.084	-0.263	-0.628	0.000	-0.167	-0.577	-0.086	-0.502	0.186	-0.469	-0.684	0.432	-0.712	0.411	-1.077
11	Synaptotagmin	Solyc07g007680	Plant synaptotagmin	0.000	-0.752	-1.165	-1.537	-0.578	-0.538	0.000	-0.256	-0.899	-0.608	-0.861	-0.336	-0.856	-1.085	-0.161	-1.015	0.096	-0.722
12	Synaptotagmin	Solyc08g021930	Synaptotagmin	0.000	-1.752	-1.560	-2.409	-2.476	-2.434	0.000	0.009	-1.559	-0.873	-1.630	-1.414	0.790	-0.965	1.465	-0.294	0.359	-0.221
13	Synaptotagmin	Solyc08g021940	Plant synaptotagmin	0.000	-0.614	-0.816	-1.427	-1.499	-0.988	0.000	-0.672	-0.634	-0.334	-1.569	-1.215	-0.489	-0.325	0.300	-0.901	0.173	0.037
14	Synaptotagmin	Solyc09g007860	Plant synaptotagmin	0.000	0.159	0.652	2.176	2.483	1.812	0.000	0.414	1.113	0.345	0.811	0.558	-0.450	-0.521	-0.061	2.046	1.832	1.017
15	Synaptotagmin	Solyc10g017590	Syt1 synaptotagmin Ca2+-dependent lipid-binding protein	0.000	0.509	0.596	0.598	0.358	0.529	0.000	0.553	0.429	0.645	0.503	0.269	-0.738	-0.581	0.282	-0.073	-0.258	-0.211
16	Synaptotagmin	Solyc10g052520	Plant synaptotagmin	0.000	-0.619	0.872	0.614	-0.547	-0.374	0.000	-0.196	-2.475	-2.242	-0.555	0.344	-2.030	-2.245	2.195	1.580	-1.415	-2.490
17	Synaptotagmin	Solyc12g009700	Plant synaptotagmin	0.000	-0.042	0.075	0.632	0.989	0.459	0.000	-0.490	-0.569	-0.550	-0.536	-0.734	-0.510	0.181	1.040	1.436	1.680	0.993
18	Synaptotagmin	Solyc12g009790	Synaptotagmin-7	0.000	0.432	0.261	0.527	1.628	1.035	0.000	-0.087	-0.450	-0.258	-0.288	-0.448	-0.339	0.413	1.269	1.208	2.222	1.406
19	Synaptotagmin	Solyc12g089330	Synaptotagmin	0.000	0.793	0.878	1.703	0.233	1.391	0.000	0.450	0.847	0.872	0.660	1.668	0.206	0.816	1.104	1.756	0.472	0.164
20	Tomosyn	Solyc02g082690	Syntaxin binding protein 5 (Tomosyn)	0.000	0.618	0.501	-0.041	0.663	0.898	0.000	0.044	-0.046	0.316	0.171	-0.042	-0.269	0.527	1.133	0.101	0.847	0.910
21	Tomosyn	Solyc06g051390	Tomosyn-like protein	0.000	0.192	-0.006	0.206	0.429	0.355	0.000	-0.029	0.152	0.054	-0.066	0.056	0.089	0.522	0.815	0.956	1.211	0.657
22	Munc13/UNC-13	Solyc01g007170	Protein unc-13 homolog C	0.000	0.278	-0.489	-0.636	-1.121	-0.795	0.000	0.363	0.253	0.386	0.402	0.009	-0.400	-0.318	-0.284	-0.802	-1.316	-0.970
23	Munc13/UNC-13	Solyc01g107740	Protein unc-13 homolog C	0.000	1.185	1.738	1.706	2.463	1.763	0.000	0.027	1.128	0.651	1.393	1.009	0.010	1.388	1.492	1.799	1.695	1.042
24	Sec1/Munc18	Solyc01g091300	Sec1-4 syntaxin-binding protein	0.000	-0.268	-0.439	-0.242	-0.059	-0.207	0.000	-0.092	0.133	-0.103	-0.177	-0.144	-0.306	-0.270	-0.008	0.266	0.434	-0.108
25	Sec1/Munc18	Solyc08g079520	Sec1-4 syntaxin-binding protein	0.000	0.607	0.685	1.220	1.131	0.617	0.000	-0.107	-0.101	0.051	-0.155	-0.331	-0.287	0.635	1.354	1.587	1.619	0.905
26	Sec1/Munc18	Solyc01g091290	Sec1-5 syntaxin-binding protein	0.000	0.124	0.073	0.283	0.633	0.562	0.000	0.335	0.487	0.641	0.512	0.567	-0.403	-0.396	0.050	-0.041	0.399	-0.139
27	AAA family ATPase	Solyc02g036340	AAA family ATPase	0.000	-0.123	-0.527	-0.982	-0.194	-0.455	0.000	-0.127	-0.263	-0.114	-0.415	-0.179	-0.132	0.100	0.493	-0.236	0.721	-0.113
28	AAA family ATPase	Solyc02g087530	AAA-ATPase	0.000	0.072	-0.019	0.200	0.631	0.597	0.000	0.587	0.948	1.021	0.817	0.798	-0.060	-0.358	-0.175	-0.177	0.377	0.003
29	AAA family ATPase	Solyc05g007470	AAA-ATPase	0.000	-0.089	-0.343	-0.492	-0.725	0.014	0.000	-0.241	-0.024	0.113	0.145	1.440	0.517	0.920	1.121	0.684	0.336	-0.623
30	AAA family ATPase	Solyc05g012190	AAA family ATPase CDC48 subfamily	0.000	-1.402	-2.434	-3.194	-2.783	-2.516	0.000	-0.778	-1.417	-1.208	-1.757	-1.772	-0.026	-0.435	-0.164	-1.295	-0.407	-0.424
31	AAA family ATPase	Solyc06g071990	AAA-type ATPase family protein	0.000	0.330	0.022	-0.516	-0.767	-1.231	0.000	-0.528	0.009	-0.116	-0.328	-0.758	0.687	1.760	1.557	1.000	0.872	0.502
32	AAA family ATPase	Solyc09g064900	AAA ATPase containing von Willebrand factor type A	0.000	-0.236	-1.185	-1.233	-1.220	-1.306	0.000	-0.176	-0.327	-0.019	-0.794	-0.313	0.318	0.499	0.345	-0.164	0.537	-0.346
33	AAA family ATPase	Solyc10g007280	AAA-ATPase	0.000	2.747	3.540	2.383	1.727	0.483	0.000	-0.987	0.271	-0.537	0.860	-0.650	0.150	4.208	4.371	3.928	1.581	1.680
34	AAA family ATPase	Solyc10g078320	AAA family ATPase CDC48 subfamily	0.000	-1.531	-1.877	-2.140	-1.875	-2.431	0.000	-0.850	-1.375	-1.421	-1.963	-2.434	-0.035	-0.546	0.407	-0.062	0.672	0.130
35	AAA family ATPase	Solyc12g055810	AAA-ATPase	0.000	-1.747	-2.690	-1.678	-0.214	-1.462	0.000	-0.224	0.675	0.675	0.095	-0.544	0.681	-0.535	-1.726	-0.589	1.065	0.225
36	AAA family ATPase	Solyc02g087540	Cell division AAA ATPase family protein	0.000	4.624	5.217	4.875	4.815	2.920	0.000	-3.605	-3.460	-0.214	1.318	-1.572	-3.930	5.180	5.991	2.302	0.331	1.168
37	BET1	Solyc06g008520	BET1 , Target SNARE coiled-coil region	0.000	-0.022	0.184	0.262	-0.059	-0.169	0.000	0.066	-0.114	-0.127	0.230	0.130	-0.072	0.062	1.107	1.043	0.278	-0.089
38	BET1	Solyc09g089630	BET1 , Target SNARE coiled-coil region	0.000	0.080	0.251	0.992	0.918	0.288	0.000	0.322	0.524	0.600	0.233	-0.413	-0.169	-0.170	0.472	0.966	1.163	0.813
39	Bet1-like protein	Solyc04g074940	Bet1-like protein At4g14600	0.000	0.497	0.523	0.784	1.090	0.536	0.000	-0.048	-0.020	-0.031	-0.041	-0.168	-0.083	0.680	1.325	1.447	1.679	0.887
40	Golgi SNAP receptor complex membe	Solyc06g065740	Golgi SNAP receptor complex member 1	0.000	0.406	0.707	0.792	0.735	1.028	0.000	-0.298	-0.121	-0.010	0.011	-0.081	-1.153	-0.244	0.586	0.396	0.246	0.269
41	Golgi SNAP receptor complex membe	Solyc01g091020	Golgi SNAP receptor complex member 1	0.000	0.170	0.403	0.497	0.672	0.177	0.000	-0.255	-0.363	-0.373	-0.322	-0.414	-0.175	0.461	1.486	1.406	1.453	0.685
42	Golgi SNAP receptor complex membe	Solyc01g091150	Golgi SNAP receptor complex member 1	0.000	0.188	0.512	0.411	0.630	0.186	0.000	-0.095	-0.713	-1.005	-0.521	-0.389	-0.397	0.120	1.707	1.729	1.397	0.462
43	Golgi SNAP receptor complex membe	Solyc05g040100	Golgi SNAP receptor complex member 2	0.000	-0.542	-0.904	-0.574	-0.346	-0.637	0.000	-0.238	-0.495	-0.306	-0.509	-0.165	0.142	0.057	0.602	0.609	0.953	-0.044
44	Golgi SNAP receptor cplx member bo	Solyc07g047720	Golgi SNAP receptor complex member bos1	0.000	0.534	0.337	0.667	0.530	-0.036	0.000	-0.027	0.359	0.391	-0.028	-0.011	0.456	1.241	1.318	1.455	1.648	0.705
45	Novel plant SNARE 11	Solyc08g077550	Novel plant SNARE 11	0.000	1.367	1.401	1.830	2.229	0.913	0.000	-0.131	-0.024	0.264	-0.586	-0.870	0.016	1.728	2.287	2.274	3.438	2.038
46	Novel plant SNARE 11	Solyc12g098950	Novel plant SNARE 11	0.000	0.018	-0.040	0.038	0.541	0.246	0.000	0.251	0.244	0.267	0.088	0.236	0.013	-0.001	0.605	0.512	1.103	0.293
47	Pentatricopeptide repeat	Solyc10g008570	Pentatricopeptide repeat-containing protein	0.000	-0.442	-0.813	-1.342	-1.320	-1.443	0.000	-0.564	-0.699	-1.228	-1.921	-2.563	-0.238	0.045	0.542	0.254	1.021	1.476
48	SNAP25 homologous protein	Solyc06g069570	SNAP25 homologous protein SNAP33	0.000	0.705	0.630	0.885	1.486	0.734	0.000	-0.018	-0.183	-0.409	-0.542	-0.344	0.210	1.156	1.857	2.221	2.839	1.541
49	SNARE associated Golgi protein	Solyc01g102530	SNARE associated Golgi protein	0.000	0.302	0.581	0.756	0.602	0.384	0.000	0.049	0.213	0.110	0.283	0.136	0.056	0.520	1.286	1.414	0.999	0.559
50	SNARE associated Golgi protein	Solyc06g064570	SNARE associated Golgi protein	0.000	0.482	0.225	0.146	0.173	0.799	0.000	0.541	-0.230	0.015	0.827	0.385	-1.683	-1.528	-0.405	-0.820	-1.530	-0.952
51	SNARE associated Golgi protein	Solyc11g040130	SNARE associated Golgi protein	0.000	-0.130	-0.289	-0.374	-0.060	-0.111	0.000	0.012	-0.341	-0.319	-0.278	-0.235	0.211	0.286	1.144	0.871	1.057	0.611
52	SNARE associated Golgi protein	Solyc08g079670	SNARE associated Golgi protein	0.000	0.837	0.682	0.806	1.524	1.166	0.000	-0.170	-0.740	-0.657	-0.417	0.058	-0.456	0.775	1.837	1.727	2.121	0.949
53	SNARE associated Golgi protein	Solyc01g080110	SNARE associated Golgi protein	0.000	-0.511	-0.334	0.290	0.518	0.209	0.000	-0.331	-0.143	-0.286	-0.289	-0.208	0.315	0.344	1.010	1.606	1.747	0.997
54	Syntaxin	Solyc01g006950	Syntaxin , t-SNARE	0.000	3.254	5.221	5.273	5.504	3.598	0.000	-0.795	-0.343	-2.560	-1.997	-0.336	-0.467	3.490	5.037	7.782	7.262	3.336
55	Syntaxin	Solyc01g056810	Syntaxin , t-SNARE	0.000	-0.348	-0.796	-1.288	-1.605	-1.295	0.000	0.180	-0.160	-0.091	-0.037	-0.031	0.504	0.212	0.768	0.120	-0.423	-0.470
56	Syntaxin	Solyc01g109750	Syntaxin ,t-SNARE	0.000	0.562	-1.136	-1.747	-2.954	-3.364	0.000	0.601	-0.806	-0.479	-0.167	-0.166	-0.090	0.015	0.389	-0.807	-2.272	-2.068
57	Syntaxin	Solyc02g085090	Syntaxin, t-SNARE	0.000	0.557	-0.283	-0.498	-1.339	-0.982	0.000	0.539	0.146	0.525	0.014	0.390	0.455	0.646	0.932	0.161	-0.297	-0.685
58	Syntaxin	Solyc06g053760	Syntaxin, t-SNARE	0.000	0.373	-0.445	-0.980	-0.387	-1.529	0.000	0.263	-0.953	-0.531	-0.398	-0.355	-0.325	-0.120	0.950	-0.218	0.203	-1.315
59	Syntaxin	Solyc07g052470	Syntaxin , t-SNARE	0.000	-0.712	-0.107	0.321	0.092	0.655	0.000	-0.138	-0.659	-0.378	0.142	0.263	-0.584	-0.943	0.891	0.821	-0.003	0.069
60	Syntaxin	Solyc10g081580	Syntaxin , t-SNARE	0.000	0.186	0.273	0.294	0.562	0.084	0.000	-0.054	-0.063	-0.191	0.006	-0.319	-0.099	0.365	1.160	1.132	1.098	0.578
61	Syntaxin	Solyc10g081850	Syntaxin , t-SNARE	0.000	0.897	1.036	1.124	1.767	1.285	0.000	0.030	0.120	-0.086	-0.136	-0.213	-0.706	0.368	1.070	1.212	1.829	1.059
62	Syntaxin	Solyc12g005580	Syntaxin , t-SNARE	0.000	-1.518	-2.685	-1.138	-2.951	-1.177	0.000	1.502	1.559	1.389	0.345	0.403	2.931	-1.255	-1.308	0.124	-0.558	1.255
63	Syntaxin	Solyc06g072760	Syntaxin , t-SNARE	0.000	0.106	0.264	-0.499	-0.809	-1.551	0.000	0.724	-0.168	0.039	-1.313	-1.457	1.018	0.436	2.169	1.098	1.994	1.357
64	Syntaxin 32	Solyc07g054140	Syntaxin 32 ,t-SNARE	0.000	1.211	1.328	1.474	1.601	0.719	0.000	0.510	0.846	0.743	0.528	0.219	-0.036	0.884	1.312	1.406	1.668	0.716
65	Syntaxin 32	Solyc08g067910	Syntaxin 32 , t-SNARE	0.000	0.403	0.253	0.556	0.731	0.371	0.000	-0.041	0.125	0.256	-0.049	-0.045	0.015	0.676	1.011	1.033	1.425	0.694
66	Syntaxin 81	Solyc03g033850	Syntaxin 81 , SNARE-complex protein Syntaxin-18 N-termi	0.000	-0.011	0.075	0.365	-0.060	-0.183	0.000	-0.128	-0.244	-0.464	-0.224	-0.395	-0.166	0.167	1.040	1.378	0.624	0.313
67	Syntaxin 81	Solyc04g071730	Syntaxin 81 ,SNARE-complex protein Syntaxin-18 N-termir	0.000	-0.078	-0.150	0.106	-0.092	-0.172	0.000	-0.206	-0.239	-0.129	-0.242	-0.399	-0.169	0.178	0.810	0.786	0.612	0.336
68	Syntaxin-51	Solyc10g081060	Syntaxin-51 ,Target SNARE coiled-coil region	0.000	0.147	-0.085	0.076	0.132	-0.090	0.000	-0.150	-0.009	-0.173	0.068	-0.062	0.241	0.755	1.049	1.206	0.939	0.490
69	Syntaxin-71	Solyc10g084210	Syntaxin-71 ,Target SNARE coiled-coil region	0.000	0.159	0.039	0.208	0.594	0.127	0.000	-0.090	-0.228	-0.022	-0.312	-0.619	-0.099	0.371	1.045	0.858	1.443	0.928
70	Syntaxin-71	Solyc11g069230	Syntaxin-71 ,Target SNARE coiled-coil region	0.000	-1.064	-0.483	-0.219	-0.464	0.313	0.000	-0.232	-0.008	0.471	0.450	0.765	0.433	-0.187	0.789	0.418	0.137	0.242
71	Syntaxin-8	Solyc07g065030	Syntaxin-8 , Target SNARE coiled-coil region	0.000	-0.420	-0.274	-0.118	-0.115	-0.407	0.000	0.033	0.162	0.028	0.201	0.091	0.112	-0.123	0.554	0.688	0.427	-0.122
72	Syntaxin-like protein	Solyc12g089150	Syntaxin-like protein ,Target SNARE coiled-coil region	0.000	-0.529	-0.097	-0.714	-1.524	-2.538	0.000	-0.413	-0.065	-0.342	-0.855	-1.930	-0.238	-0.127	0.624	0.122	-0.262	-0.510
73	Syntaxin-like protein	Solyc06g062360	Syntaxin-like protein , t-SNARE	0.000	1.154	0.558	0.617	0.020	0.649	0.000	0.788	1.937	0.880	0.969	0.600	-0.052	0.973	-1.063	0.457	-0.395	0.299
74	Syntaxin-like protein	Solyc08g005200	Syntaxin-like protein , t-SNARE	0.000	-0.097	-0.239	-0.276	-0.209	-0.461	0.000	-0.082	0.069	-0.034	-0.166	-0.649	0.057	0.256	0.621	0.545	0.642	0.507
75	Syntaxin-like protein	Solyc08g076540	Syntaxin-like protein ,t-SNARE	0.000	1.000	2.083	1.808	2.025	1.392	0.000	-0.285	-0.155	0.149	0.280	-0.035	-0.628	0.906	2.466	1.728	1.722	1.071
76	Syntaxin-like protein	Solyc11g066910	Syntaxin-like protein , t-SNARE	0.000	0.025	-0.526	-1.124	-1.624	-0.968	0.000	0.080	-0.064	-0.039	0.621	0.005	0.048	0.207	0.485	-0.374	-1.756	-0.520
77	Transmembrane protein 41B	Solyc08g076670	Transmembrane protein 41B ,SNARE	0.000	0.521	0.850	0.850	0.813	1.184	0.000	0.696	1.160	0.891	0.962	-0.050	-0.554	-0.508	-0.011	0.147	-0.070	0.941
78	Transmembrane protein 41B	Solyc06g065400	Transmembrane protein 41B ,SNARE associated Golgi prot	0.000	0.755	0.904	0.867	1.448	1.038	0.000	0.063	0.289	-0.187	0.026	-0.066	0.010	0.911	1.488	1.777	2.055	1.374
79	Transmembrane protein 41B	Solyc11g018620	Transmembrane protein 41B ,SNARE associated Golgi prot	0.000	-0.217	-0.675	-0.422	-0.113	0.109	0.000	-0.205	-0.434	-0.519	-0.703	-0.162	-0.038	0.156	0.598	0.762	1.172	0.494
80	T-snare	Solyc09g055760	T-snare	0.000	-0.003	-0.261	-0.036	0.271	-0.145	0.000	-0.086	0.318	0.285	-0.047	-0.265	0.475	0.772	0.774	0.872	1.421	0.869
81	Vesicle transport v-SNARE 11-like	Solyc03g115760	Vesicle transport v-SNARE 11-like	0.000	-0.294	0.023	0.121	-0.052	-0.196	0.000	-0.151	-0.103	-0.314	0.027	-0.118	-0.339	-0.267	0.678	0.818	0.215	-0.151
82	Vesicle transport v-SNARE 11-like	Solyc05g013050	Vesicle transport v-SNARE 11-like	0.000	-0.223	-0.399	-0.112	0.032	0.279	0.000	-0.043	-0.179	-0.102	-0.052	-0.068	-0.012	0.029	0.643	0.696	0.705	0.603

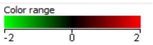

Supplement: Supplementary file 1 [file life-10-00273-s001.zip › supplementary for XML/Supplementary Table S2.pdf]
