# Supplementary material for: Expression Kinetics of Regulatory Genes Involved in the Vesicle Trafficking Processes Operating in Tomato Flower Abscission Zone Cells during Pedicel Abscission
Source: Life (Basel). 2020 Nov 6;10(11):273. doi: 10.3390/life10110273 (PMC7694662; doi:10.3390/life10110273)
Supplement: Supplementary file 1 [file life-10-00273-s001.zip › supplementary for XML/Table S3 .pdf]

life

Supplementary materials of Expression Kinetics of Regulatory Genes Involved in the Vesicle Trafficking Processes Operating in Tomato Flower Abscission Zone Cells during Pedicel Abscission

Table S3. List of primers and their sequences used in the qRT-PCR assay.

No	Primer Name	Gene Bank ID	Solyc ID	Primer Sequence (5' to 3')	Tm (°C)
**1**	Actin_F	AB199316	Solyc03g078400	GTGTTGGACTCTGGTGATGG	60
**2**	Actin_R			GTAGTCAAGAGCCACATAAGC	60
**3**	TAPG1_F	U23053	Solyc02g067630	GCAGTGAAACTTGATTGTAGC	56
**4**	TAPG1_R			CCATTCTTGTGATAGTATACAC	56
**5**	TAPG4_F	U70481	Solyc12g096750	GAACATCAGCTACAGAAATCG	60
**6**	TAPG4_R			ACCAGAAGCTCTTCCTCCAG	60
**7**	Cel1_F	U13054	Solyc08g081620	GGATTAATGCCAGAAAGTAGC	60
**8**	Cel1_R			GTAACTCCTCCTATTCCAGC	60
**9**	TPRP-F1_F	X57076	Solyc07g043000	GCACAACTATTAGACTCAAGC	60
**10**	TPRP-F1_R			TGCCTTCAACTATGACAATGC	60
**11**	KD1_F	AF375969	Solyc06g072480	CTCACTCACAATGGATCAACC	60
**12**	KD1_R			GGAGTGGAAGTAGAAGTAGG	60

Publisher's Note: MDPI stays neutral with regard to jurisdictional claims in published maps and institutional affiliations.

© 2020 by the authors. Submitted for possible open access publication under the terms and conditions of the Creative Commons Attribution (CC BY) license (http://creativecommons.org/licenses/by/4.0/).
